# Determination of production regions for pollution-free Chinese medicinal materials by geographic information system: *Achyranthes bidentata* (Niu Qi) in Tianjin as an example

**DOI:** 10.1186/1749-8546-9-25

**Published:** 2014-09-19

**Authors:** Cai-xiang Xie, Huan Wang, Lin-fang Huang, Hui Li, Jing-yuan Song

**Affiliations:** 1 The Key Laboratory of Bioactive Substances and Resources Utilization of Chinese Herbal Medicine, Ministry of Education, Institute of Medicinal Plant Development, Chinese Academy of Medical Sciences & Peking Union Medical College, Beijing 100193, P. R. China

## Abstract

**Background:**

The land and ecology suitability for producing pollution-free Chinese medicinal materials should be evaluated based on Geographic Information System (GIS). This study aims to determine production regions for pollution-free *Achyranthes bidentata* in Tianjin as a case to illustrate the workflow based on GIS.

**Methods:**

The slopes, land usage, residential areas and roads were selected to evaluate the land suitability, to avoid the potential pollution sources. The ecology suitability evaluation was performed based on the soil type and nine climate factors, such as active accumulated temperature, mean January temperature, mean July temperature, the lowest temperature in January, the highest temperature in July, mean annual temperature, sunshine duration, relative humidity, annual precipitation, affecting the natural growth of *A. bidentata*.

**Results:**

The best production regions for pollution-free *A. bidentata* in Tianjin, with a total area of approximately 575 km^2^, were found in Jinghai County, Ninghe County, Wuqing District, and Dagang District.

**Conclusion:**

This study illustrated a workflow based on GIS for determining the production regions in Tianjin for pollution-free *A. bidentata.*

## Background

Good Agricultural Practices (GAPs) focused on production of the pollution-free Chinese medicinal materials for the food and drug safety [1]. It is crucial to determine appropriate regions for the growth of Chinese medicinal materials to avoid existing or potential pollution sources. Both land suitability and ecology suitability should be considered to determine the production regions.

There are many applications in determining the space location by Geographic Information System (GIS) [2-4]. GIS is designed to work with data referenced by spatial or geographic coordinates. GIS can operate all database with spatial properties, *i.e.*, the climate database and land usage database, such as query and statistical analysis, with the ability to see how data relates in space and time. The maps produced with GIS are useful for showing places and the events that occur there. Therefore, GIS are used in this paper to determine the production regions for pollution-free Chinese medicinal materials.

**Figure 1:**
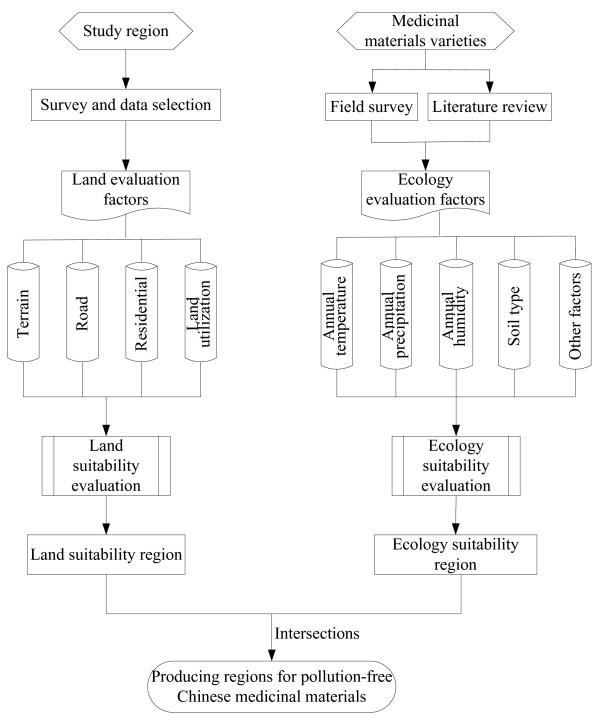
Technology flow chart for determination of production regions of pollution-free Chinese medicinal materials.

*A. bidentatae* has been embodied in Chinese Pharmacopoeia 2010 and listed as one of very important Chinese medicine material by State Administration of Traditional Chinese Medicine of the People’s Republic of China. *A. bidentatae* is mainly cultivated in Henan Province and widely distributed in Tianjin, Shandong, Shanxi, Hebei, Guizhou, Yunnan, and other provinces (or cities) [5]. Compared with the previous study [6-11], the land suitability was firstly taken into account in this paper with ecological suitability to assure production regions not only suitable for *A. bidentata*, but also away from pollution sources [12,13]. The slopes, land usage, residential areas and roads in Tianjin were selected to evaluate the land suitability [14,15]. The factors in ecology suitability evaluation were soil type and nine climate indicators, including active accumulated temperature, mean January temperature, mean July temperature, minimum January temperature, maximum July temperature, mean annual temperature, sunshine duration, relative humidity, and annual precipitation, shown in Figure 1.

This study aims to determine the production regions for pollution-free *A. bidentata* in Tianjin, as a case to illustrate the workflow based on GIS.

## Methods

### Study area and data sources

In Tianjin, the annual average temperature is 11.4°C to 12.9°C, the average temperatures in January and July were respectively from -3°C to -5°C and from 26°C to 27°C, the annual average precipitation was 520 mm to 660 mm, the annual average wind speed was 2 to 4 meters per second, and annual sunshine duration was 2,500 hours to 2,900 hours [16].

The data (Additional file 1) for the land suitability evaluation, such as slopes, roads, residential areas and land usage, were obtained from the Data Center for Resources and Environmental Sciences Chinese Academy of Sciences (RESDC). The slope data were obtained from Tianjin 1:100,000 DEM data, the residential regions were from 1:100,000 land-use map, the climate data for the ecology suitability evaluation were from National Weather Bureau, which were ed into the raster data of 1 square kilometers based on the GIS [17].

### Evaluation of land suitability

#### Evaluation factors

Evaluation factors should follow several principles: superiority (*e.g.*, factors which has larger affection on the land suitability), stability (*e.g.*, factors which have long-term stable influence on production regions) and accessibility [18,19]. Therefore, the slopes, land usage, residential areas and roads were selected to evaluate the land suitability. The automobile exhaust in highway and residential region could be the potential pollution sources, the landform was closely related to the cultivation costing, and the land usage data implied that whether certain land can be used to cultivate.

#### Grading rules of the land status

Production regions should be far away from the pollution sources *e.g.*, roads and residential areas, and suitable for cultivation. Farmlands should not be considered, because they are possibly polluted by pesticides and chemical fertilizers. Furthermore, the land with a slope exceeding 25° would not be suitable for crop cultivation, since many Chinese herbal medicines only grow better in regions with slope under 15° [20].

Each factor was graded on a 10-point scale by the Delphi method for agreement among experts [21]. The higher the scores, the more suitable the land would be for producing region. With reference to the standards in pollution-free agriculture, the grading rules of single factors were listed in Table 1.

**Table 1:** Grading rules of single factors for land suitability

**Level**	**Rating score**				
**Assessment factors**	**10**	**8**	**6**	**4**	**2**
Distance from road (m)	> 3000	3000–2000	2000–1000	1000–500	< 500
Distance from residential area (m)	> 5000	5000–3000	3000–2000	2000–1000	< 1000
Land slope (°)	0–5	5–10	10–15	15–20	> 20
Land usage status	Tidal flats and wasteland	Meadow	Cultivated land	Waters and residents not considered	

#### Land suitability evaluation process

Evaluation factors were graded into five levels, the land with a higher grade was more suitable for producing region. Comprehensive multi-factor evaluation of land production proceeded on the basis of single-factor evaluation. Analytic Hierarchy Process (AHP) could organize and analyze complex decisions, based on mathematics and psychology, for structuring a decision problem, representing and quantifying its elements, relating those elements to overall goals, and evaluating alternative solutions. AHP was used to calculate the corresponding weights of these factors [22]. The land suitability map in Tianjin was generated by GIS software.

##### Single factor evaluation

**Figure 2:**
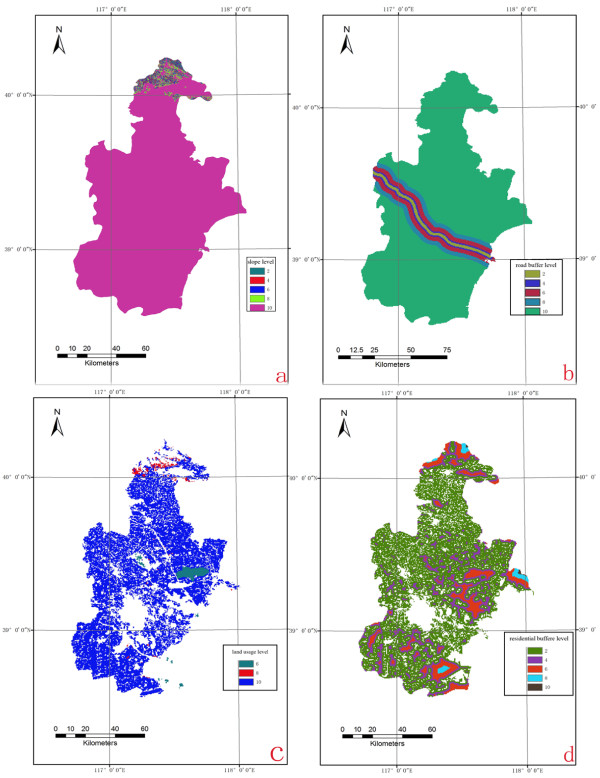
**Single-factor evaluation for land suitability. (a)** Slope; **(b)** Road distance; **(c)** Land usage; and **(d)** Residential area.

Flat land is appropriate for the production regions due to the smaller the slope, the higher grade the land is. The grade map based on the land slope in Tianjin was shown in Figure 2a. The land far away from roads had higher score due to free of automobile exhaust. The re-classified map of Tianjin based on the distance from the road was shown in Figure 2b. The lands in Tianjin were classified into four grades in Table 1. The tidal flats and wastelands were the most suitable for production regions following by the meadow and cultivated land; water bodies and residential areas should not be considered because of their situation. The re-classified map based on the land usage factor was shown in Figure 2c. Considering the pollution caused by human, the optimum production regions should be away from residential region. The grade map in Tianjin was shown in Figure 2d.

##### Multi-factors evaluation

These factors had various effects on the land suitability. Hence, AHP was used to calculate the index weights. The judgment matrix A had the following characteristics:

(1) $$A={\left(a_{\mathrm{ij}}\right)}_{n\times n},\;a_{\mathrm{ij}}>0,\;a_{\mathrm{ij}}=\frac{1}{a_{\mathrm{ji}}}$$

(2) $$\mathrm{CI}=\frac{\lambda -n}{n-1},\;\mathrm{CR}=\frac{\mathrm{CI}}{\mathrm{RI}}$$

where λ is the maximum eigenvalue of matrix A, n is the order of A, CI is the consistency check index, and RI is the random consistency index. The weights of slopes, residential areas, roads, and land status were 0.21, 0.19, 0.27, and 0.33, respectively.

(3) $$V=\sum_{i=1}^{n}\left(W_{i}\times P_{i}\right)$$

The equation (3) was adopted as comprehensive evaluation model, where *V* is the value of evaluation results; *W*_*i*_ is the weight value of the *i-*th indicator; *P*_*i*_ is the grade score of the *i-*th indicator; and *n* is the number of indicators.

### Evaluation of ecology suitability

#### Evaluation factors

Referring to the assessment standards in agriculture, ten ecological factors were included (Table 2): accumulated temperature exceeding 10°C, annual average temperature, average temperature in January, the lowest temperature in January, average temperature in July, the highest temperature in July, annual average relative humidity, annual average sunshine duration, annual average precipitation, and soil type. The climate data in the above regions were obtained from the spatial database and climate database [23], which could be considered as the optimum ecological suitability of *A. bidentata*.

**Table 2:** **Ecological suitability of** ***A. bidentata***

**Climate factors**	**AT (°C)**	**AAT (°C)**	**MTJA (°C)**	**TLTJ (°C)**	**MTJ (°C)**	**THTJ (°C)**	**AARH (%)**	**AASH (h)**	**AAP (mm)**
	1200.7 ~ 8254.0	12.4 ~ 28.0	-3.8 ~ 17.4	-9.3	14.9 ~ 29.2	34.1	60.4 ~ 85.0	1047 ~ 2190	521 ~ 1803
Soil types	Crimson soil, red, yellow soil, yellow brown soil, and brown soil, brown, dark brown.								

Note: AT (accumulated temperature exceeding 10°C); AAT (annual average temperature); MTJA (mean temperature in January); TLTJ (the lowest temperature in January); MTJ (mean temperature in July); THTJ (the highest temperature in July); AARH (annual average relative humidity); AASH (annual average sunshine hours); AAP (average annual precipitation).

#### Ecology suitability evaluation process

**Figure 3:**
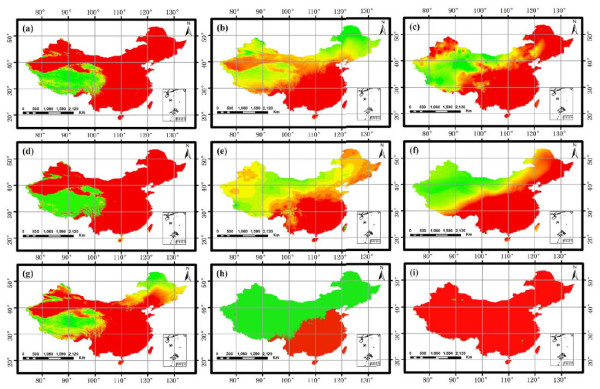
**Standardization results of evaluation factors. (a)** mean July temperature. **(b)** mean January temperature. **(c)** relative humidity. **(d)** active accumulated temperature. **(e)** sunshine duration. **(f)** annual precipitation. **(g)** mean annual temperature. **(h)** the lowest temperature in January. **(i)** the highest temperature in July.

Evaluation factors were firstly standardized by the mean absolute deviation formula

(4) $$Z_{\mathrm{if}}=\frac{x_{\mathrm{if}}-m_{f}}{S_{f}}$$

(5) $$m_{f}=\frac{1}{n}\left(x_{1f}+x_{2f}+\cdots +x_{\mathrm{nf}}\right)$$

(6) $$S_{f}=\frac{1}{n}\left(\left|x_{1f}-m_{f}\right|+\left|x_{2f}-m_{f}\right|+\cdots \cdots +\left|x_{\mathrm{nf}}-m_{f}\right|\right)$$

where *x*_1*f*_, *x*_2*f*_, …, *x*_*mf*_ is the measurements of variables *f*, *n* is the number of the measurements, *m*_*f*_ is the average for *f*, *S*_*f*_ is the average of the absolute deviations for *f*, *Z*_*if*_ is the normalized value of *f*, the normalized results of the ten ecological factors were shown in Figure 3. Climate similarity evaluates whether the weather was suitable for *A. bidentata* with statistical distances [24-26]. The smaller the distance, the higher similarity the climates were.

### Software

ArcGIS 10.2, supplied by Esri China Information Technology Co. Ltd., was a software used in the study, and could operate all database with spatial properties, the operated database by GIS in this paper were the climate database and land usage database [23]. The preliminary data processing was vital to complete the implementation. Because there were various data formats, it was necessary to convert various data formats into the same formats suitable for GIS.

## Results

**Figure 4:**
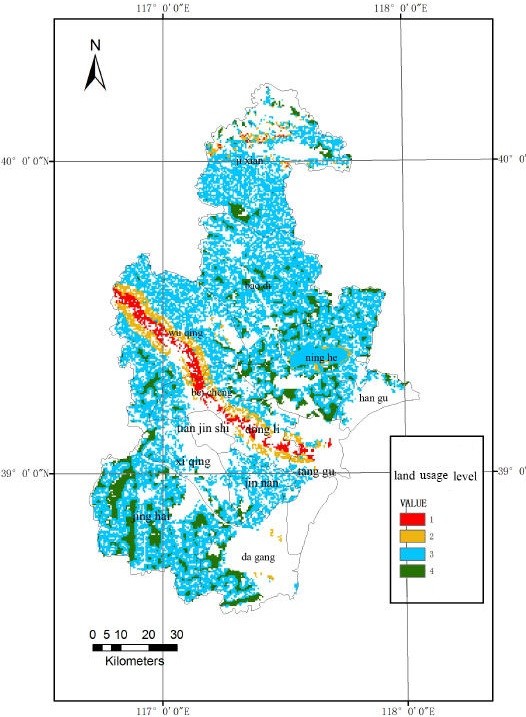
Evaluation grade of land suitability.

**Figure 5:**
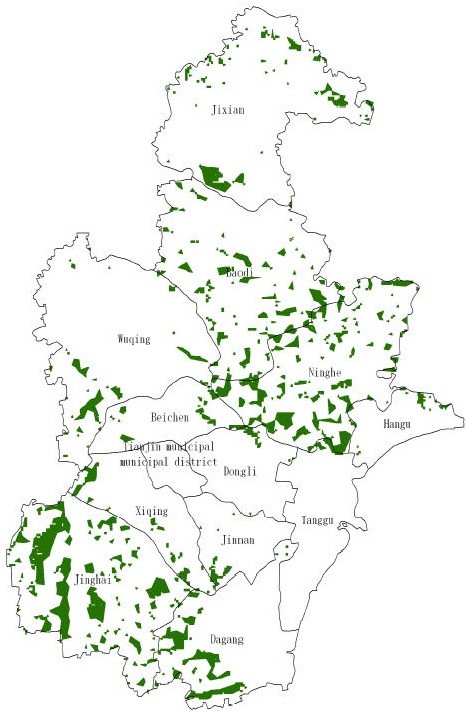
Optimum land patches for land suitability.

The final land suitability evaluation results were shown in Figure 4, the green portions represent the optimum production regions for pollution-free *A. bidentata*, the blue portions are the secondly suitable regions, the yellow and the red portions are the unsuitable regions. The optimum regions for land suitability were shown in Figure 5, which are mainly in Jinghai County, Ninghe County, Baodi District, Dagang District, and other suburban counties of Tianjin, with a total of 513 patches approximately 836 km^2^.

**Figure 6:**
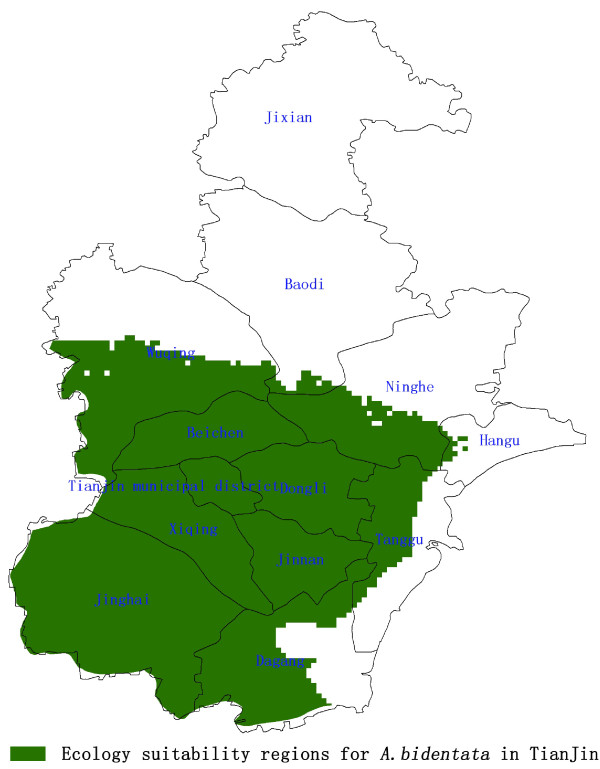
**Ecology suitability region for *A. bidentata *** **in Tianjin.**

**Figure 7:**
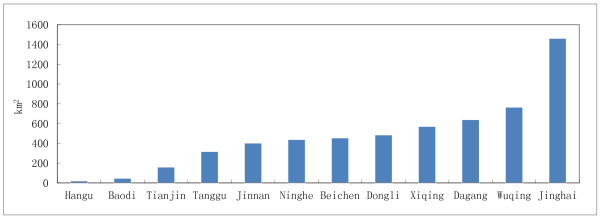
**Quantities distribution of ecology suitability region for *A. bidentata *** **in Tianjin.**

The optimum ecological suitability zones for *A. bidentata* in Tianjin were shown in Figure 6, which area is 5766 km^2^, in Jinghai County is 1462 km^2^, in Wuqing is 766 km^2^, in Dagang District is 640 km^2^, other regions are in Dongli District, Beichen District, Ninghe, Jinnan District, Tanggu District (Figure 7).

**Figure 8:**
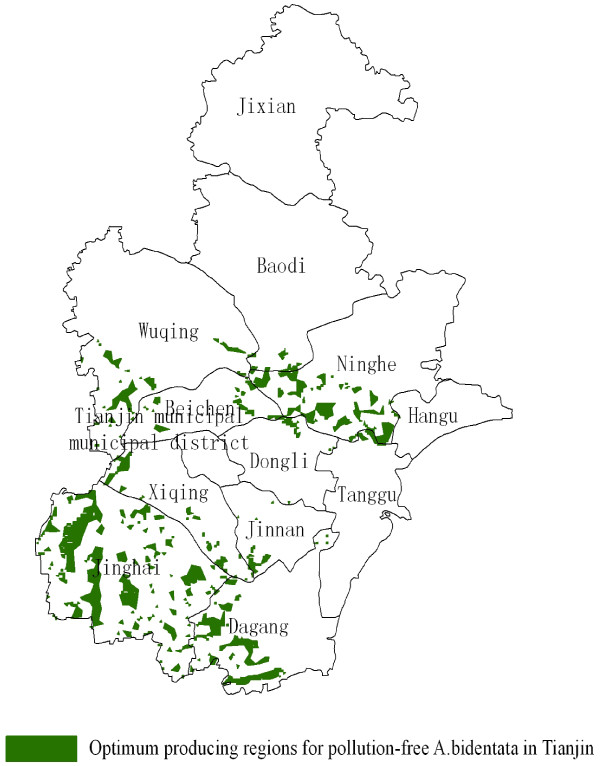
**Optimum production regions for pollution-free *A. bidentata *** **in Tianjin.**

Therefore, the production regions for pollution-free *A. bidentata* should meet the criteria of both ecology suitability and land suitability. The regions should be in the overlapping zones of the optimum ecology suitability regions and the best areas of land suitability. The optimum ecological regions for *A. bidentata* were measured to be 5766 km^2^, mainly located in the southern of Tianjin. Moreover, the area of land suitability was measured to be 836 km^2^. The most suitable production regions for pollution-free *A. bidentata* in Tianjin were in the overlapping zones, which area was measured to be 575 km^2^ and mainly located in the counties of Jinghai, Ninghe, Dagang District, and Wuqing District (Figure 8).

## Conclusion

This study illustrated a workflow for determining the production regions of pollution-free *A. bidentata* in Tianjin based on GIS.

## Abbreviations

GAPs: Good agricultural practices; GIS: Geographic information system.

## Competing interests

The authors declare that they have no competing interests.

## Authors’ contributions

SJY conceived and designed the study. XCX, WH, HLF and LH performed the data analysis. XCX and LH wrote the manuscript. All authors read and approved the final version of the manuscript.

## Supplementary Material

Additional file 1 Land utilization. Click here for file
